# Vision, Its Optical Defects and Adaptation of Spectacles

**Published:** 1875-11

**Authors:** 


VISION—ITS OPTICAL DEFECTS AND ADAPTATION OF SPECTACLES. By C. S. Fenner, M.D. Published by Lindsay & Blakiston, Philadelphia. Book; cloth; pp. 289.

This is the first edition issued, and the author in the preface says: "In preparing this work for the press the endeavor has been made to give in a concise and popular, yet comprehensive form, *a resume* of our present knowledge of *physiological optics*, and the defects of the eye as an optical instrument." It is divided into three parts. The first is devoted to light; second, to physiological optics; third, to errors in refraction and defects of accommodation. It is well written, neatly arranged, and no doubt will be well received by the profession.

